# Extracellular histone H1 is neurotoxic and drives a pro-inflammatory response in microglia

**DOI:** 10.12688/f1000research.2-148.v1

**Published:** 2013-07-08

**Authors:** Jonathan D Gilthorpe, Fazal Oozeer, Julia Nash, Margarita Calvo, David LH Bennett, Andrew Lumsden, Adrian Pini

**Affiliations:** 1 MRC Centre for Developmental Neurobiology, King’s College London, London, SE1 1UL, UK; 2 Department of Pharmacology and Clinical Neuroscience, Umeå University, Umeå, S-901 87, Sweden; 3 Wolfson Centre for Age Related Diseases, King's College London, London, SE1 1UL, UK; 4 Nuffield Department of Clinical Neurosciences, University of Oxford, John Radcliffe Hospital, Oxford, OX3 9DU, UK

## Abstract

In neurodegenerative conditions and following brain trauma it is not understood why neurons die while astrocytes and microglia survive and adopt pro-inflammatory phenotypes. We show here that the damaged adult brain releases diffusible factors that can kill cortical neurons and we have identified histone H1 as a major extracellular candidate that causes neurotoxicity and activation of the innate immune system. Extracellular core histones H2A, H2B H3 and H4 were not neurotoxic. Innate immunity in the central nervous system is mediated through microglial cells and we show here for the first time that histone H1 promotes their survival, up-regulates MHC class II antigen expression and is a powerful microglial chemoattractant. We propose that when the central nervous system is degenerating, histone H1 drives a positive feedback loop that drives further degeneration and activation of immune defences which can themselves be damaging. We suggest that histone H1 acts as an antimicrobial peptide and kills neurons through mitochondrial damage and apoptosis.

## Introduction

All of the major neurological diseases and trauma to the central and peripheral nervous systems carry the same cytopathological features, namely neuronal death and reactive gliosis. These differential responses occur simultaneously within the same milieu but why this happens is poorly understood. The degenerating nervous system is not a non-specific killing field.

The observations described here derive from experiments designed to identify axonal chemorepellents in which explants of degenerating adult rat brain were co-cultured with embryonic brain explants in collagen gel matrices. Unexpectedly, we found that adult brain causes diffusion-mediated degeneration of embryonic axons and have shown that the degenerating adult brain releases histone proteins. Histone H1, but not core histones, selectively kills embryonic cortical neurons and causes microglial cells to become reactive.

Histones are highly basic proteins that are present in the nucleus where they form major components of eukaryotic chromatin and function to regulate transcription. The core histones, H2A, H2B, H3 and H4 have relatively similar structures containing a histone fold domain and together form the histone octamer. The winding of genomic DNA around this octamer forms the basic unit of DNA packaging termed the nucleosome. Members of the more divergent histone H1/H5 family are structurally unrelated to core histones and function to link nucleosomes. It has usually been assumed that histones are restricted to the nucleus but a growing body of evidence indicates that histones, histone-DNA complexes and histone-derived peptides have cytoplasmic and extracellular locations and actions ^1^.

Core and linker histones and nucleosomes have been detected both at the cell surface ^2– 6^ and in the cytoplasm ^7, 8^ and have also been linked to apoptosis ^7– 11^. Several lines of evidence support the idea that extracellular histones may contribute to human disease mechanisms. Anti-histone antibodies are present in vascular dementia and pre-senile Alzheimer’s Disease (AD) ^12^. In systemic lupus erythematosus (SLE), many different nuclear antigens are released and provoke high titres of destructive anti-histone autoantibodies ^8^. Indeed, a significant proportion of patients with SLE have associated neurodegeneration. More recent evidence indicates that extracellular histones can mediate endothelial cell death and sepsis ^13^, and cause thrombocytopenia through platelet aggregation ^14^.

## Materials and methods

### Animals

Sprague Dawley and Wistar rats were obtained from Charles River and kept on a 12-h light-dark cycle with food and drinking water provided *ad libitum.* Animals were killed under Schedule 1 by cervical dislocation conforming to British Home Office Regulations. For neuronal cultures cerebral cortices were removed from embryonic day (E) 15–17 Sprague Dawley rat embryos and adult brain explants were obtained from the mother.

For microglial cultures the mother and her litter were housed in one cage and post-natal day 3 (P3) pups were killed under British Home Office Schedule 1 regulations by rapid decapitation.

For substratum experiments mouse cortical neurons were prepared from E16.5 CBA x C57BL/6 mice (bred in house Umeå University) in accordance with approval granted by Umeå University Ethical Committee for Animal Experimentation (Permit Number: A113-11). Animals were maintained as part of a breeding colony in a designated facility on a 12-h light-dark cycle with food and drinking water provided *ad libitum.* Neuronal isolation was performed after the mother had been sacrificed by cervical dislocation (the morning a vaginal plug was discovered was determined to be E0.5).

All animal experiments were designed and performed according the mandated principles of reduction, refinement and replacement.

### Tissue culture

Cortical explants were dissected into pieces of about 200–400 μm ^2^ (embryonic) and 500–1200 μm ^2^ (adult) using fine tungsten needles and kept on ice-cold minimum essential medium (MEM, Gibco UK). For dissociated cultures embryonic cortices were also cut into small pieces and dissociated with the Papain Dissociation System (Worthington Biochemicals) according to the manufacturer’s instructions. Neurons were plated on 13 mm diameter glass coverslips coated first with poly-D-lysine (10 μg/ml in PBS) followed by laminin (10 μg/ml in PBS) (Gibco) and cultured at 37°C in a humidified 8% CO _2_ (v/v) atmosphere for 24–48 hrs in Neurobasal medium plus 1% (v/v) Antibiotic-Antimycotic (Gibco). To make conditioned media, the meninges were removed from adult and embryonic brains and were then cut into small pieces (~2 mm ^3^) and kept on ice-cold MEM.

Collagen was prepared by mixing 90 µl of filtered rat tail collagen as described in ^15^ with 10 µl of 10× concentrated Dulbecco's Modified Eagle Medium (DMEM, Gibco), which was kept on ice until required ^15, 16^, and set by mixing with 2–3 μl of 7.5% (w/v) sodium bicarbonate (Gibco). Explants were placed on 35 mm Falcon dishes, excess liquid aspirated with a fine glass capillary tube and covered with 30 μl of the setting collagen solution. Co-cultures of adult and embryonic cortex were now positioned ~0.5–1 mm apart and lectin-coated agarose beads (Vector Laboratories) (1–2 μl) injected between them when required. Co-cultures and dissociated cells were incubated for 24–48 hrs in DMEM plus 1% (w/v) Antibiotic-Antimycotic (Gibco) at 37°C in a humidified 8% (v/v) CO _2_ atmosphere and examined by phase contrast microscopy (Nikon T800 and Lucia software).

Adult conditioned medium was made by incubating two chopped cortices per 20 ml culture medium while for embryonic conditioned medium four E15 whole brains per ml were used. Incubations were performed for 48 hrs as above but the medium was replenished after 24 hrs. Conditioned media were centrifuged for 3 mins at 900g and stored at -20°C. Careful checks were made to ensure that pH remained physiological.

### Protein purification

Histones and other proteins were isolated and identified from adult and embryonic conditioned media. Affinity chromatography columns were constructed using agarose beads as the solid phase to which *Sambucus nigra* lectin (Vector Laboratories, 3 mg lectin/ml gel) had been covalently bound. Columns were loaded with 5 ml of conditioned media and incubated for 1 hr at room temperature before being washed with 5 mls of PBS and eluted with 5 mls of PBS and alpha-lactose (500 mM) followed by 5 mls PBS and 2% (w/v) acetic acid according to the manufacturer’s instructions. Elution was by gravity (1 ml column) or by pump (10 mls column 0.4 ml/min). Eluates were concentrated in two steps, first using 5,000 NMWL (nominal molecular weight limit) 15 ml filters (Microcon, Millipore) centrifuged at 900g at room temperature until 2 mls remained. These aliquots were further concentrated using 10,000 nominal molecular weight limit NMWL Microcon 1.5 ml filters centrifuged at 13,000g at room temperature until dry. Material was reconstituted from the membrane filters in 10–40 mls of sample buffer made according to Laemmli ^17^ and stored at -20°C.

Denaturing gel (Laemmli) electrophoresis was performed using 10% (w/v) SDS-polyacrylamide gels (SDS-PAGE). Samples were denatured prior to loading and electrophoresis was performed at a constant current of 25 mA, using a Tris-glycine buffer system with SDS (192 mM glycine (Sigma), 25 mM Trizma base (Sigma) and 0.1% SDS (Fisher Scientific)). Gels were stained with R250 Coomassie Brilliant Blue (1g Coomassie in 50% (v/v) methanol/10% (w/v) acetic acid) and destained with 12% (v/v) methanol in 7% (w/v) acetic acid for 1 minute. Molecular weight markers were run with all gels. Bands of interest were excised from the gels and stored at -20°C until sequenced.

### Sequencing

Coomassie-stained bands resolved by SDS-PAGE were excised and digested with 200 ng trypsin (Promega) for 4 hrs at 37°C. The resultant peptides were separated by reverse-phase chromatography (Vydac C18 0.8 × 150 mm) and were analysed by Matrix-assisted laser desorption/ionization-time of flight (MALDI TOF) mass spectrometry using an Applied Biosystems 4700 Proteomics Analyser. Peptide masses were searched against the NCBI (National Center for Biotechnology Information) non-redundant database using Protein Prospector MS-FIT software (University of California San Francisco).

### Histone application

Human recombinant histones H1, H2A, H2B, H3 and H4 were obtained from New England Biolabs. Additionally, histone H1 purified from calf thymus, and *Xenopus laevis* recombinant histones H2A, H2B, H3 and H4 were obtained from Millipore. Histones were diluted in culture media as indicated.

### Cortical neurons and luciferase assay

Primary cortical neurons were seeded onto poly-D-lysine and laminin (both 10 μg/ml) coated black 96-well plates (Greiner) at 3 x 10 ^4^ cells per well in 100 μl. Cells were incubated for 48 hrs in Neurobasal medium supplemented with Glutamax, B27 and 1% (v/v) Antibiotic-Antimycotic (all Gibco) and then treated with histones at 0, 25, 50, 100 and 200 nM for a further 24 hrs. Histone-induced cell death was determined by the CytoTox-Glo™ Cytotoxicity Assay (Promega). Briefly, degenerating cells release intracellular proteases that cleave acetylaminofluorene (AAF) to liberate aminoluciferin, which is a substrate for luciferase. This reaction generates luminescence proportional to the number of dead cells ^18^ and was measured using a Promega GloMax 96 luminometer. Readings were made within 24 hrs to prevent false negatives due to protease degradation.

### Substrate outgrowth and survival assay

Mouse cortical neurons were prepared from E16.5 CBA x C57BL/6 mice using a similar protocol to rat except 0.05% (w/v) trypsin digestion for 10 min at 37°C in Hank’s buffered saline (HBS, Gibco) was used instead of papain. Cells were plated at 3 x 10 ^3^ cells per well in 96-well plates in Neurobasal media supplemented with 2% (v/v) NS21 supplement 0.25 mM L-glutamine and 10 µg/ml gentamicin ^19^. Wells had been previously coated with poly-D-lysine, histone H1, or histone Type VIII-S (arginine rich fraction of calf thymus histone, predominantly histone H3, Sigma). Cells were grown for 72 hrs and the number of living neurons was estimated using calcein acetoxymethyl ester (AM) staining. Cells were incubated with 1.1 nM calcein AM for 30 min at 37°C in a humidified incubator. Hoechst 33342 (16 µM) (Molecular Probes, Invitrogen) was added to counterstain the nucleus of both live and dead cells. Haemoglobin (15 mg/ml) was then added to quench fluorescence generated by the culture medium and the plate was imaged using a TROPHOS Plate RUNNER HD cell fluorescence imaging instrument. Total cell fluorescence from calcein AM, a measure of metabolic activity from live cells, was determined using the Tina analysis package (Trophos).

### Cortical astrocyte culture

Cortical astrocytes were cultured from 2–3 days post-natal rat pups. Two cortices were removed and cut into small pieces (~2mm ^2^) before being digested with 5 ml of 0.2% (w/v) trypsin, BSA, DNase I (Sigma) in Earle’s buffer (Worthington) at 37°C for 20 mins. Digestion was terminated by addition of an equal volume of DMEM followed by centrifugation for 5 mins at 1,500 rpm. The pellet was re-suspended in 10 ml of DMEM with 10% (v/v) FCS and 1% (v/v) penicillin and streptomycin (Gibco) which was divided between two 5 ml flasks coated with poly-L-lysine (0.5 mg/ml Sigma). Flasks were incubated as described above for 10 days with media changed at 48 and 96 hrs. Cells were passaged and plated on poly-D-lysine coated coverslips for a further 6 days in 4-well plates prior to treatment with histones and staining with antibodies.

### Immunohistochemistry

Cells were fixed in 4% (w/v) paraformaldehyde (PFA) and incubated for 1 hr with anti-glial acidic fibrillary protein GFAP rabbit polyclonal antibody (Z0334 Dako) diluted at 1:2500 in PBS or cleaved caspase 3 antibody (Abcam) diluted 1:500 in PBS. Cells were washed and incubated for 1 hr in polyclonal anti-rabbit TRITC (Dako) diluted 1:2500 (for GFAP) or goat anti rabbit polyclonal Alexa Fluor 488 (Invitrogen) for caspase 3 in PBS prior to washing and mounting. Some neuronal cultures were incubated with 15 μM propidium iodide (Invitrogen) for 10 mins before fixing and with 0.5 μM DAPI (4′, 6-diamidino-2-phenylindole) (Sigma) for 5 mins after fixing during washing with PBS.

### Primary microglia cell culture

Mixed glial cultures were made from explants of cerebral cortices of P3 (3 day post-natal) Wistar rats as described previously ^20, 21^. Briefly, after mechanical and chemical dissociation cells were plated in DMEM 10% (v/v) fetal bovine serum at a density of 500,000 cells/ml and cultured at 37°C in humidified 5% (v/v) CO _2_/95% (v/v) air. Reagents were obtained from Invitrogen. Confluence of the mixed glial culture was achieved after 5 days *in vitro*. Cultures were gently shaken and the floating cells were pelleted and sub-cultured. This method resulted in 96–99% purity as assessed by staining with the rabbit anti-ionized calcium binding adaptor molecule 1 (Iba1) polyclonal antibody at 1:1000 (019-19741, Wako Chemicals, Japan) and 4′, 6-diamidino-2-phenylindole (DAPI).

### Chemotaxis assay

Chemotaxis was assessed using a Boyden ^22^ chamber (Neuroprobe, Bethesda, MD). Polycarbonate filters (5 μm pore diameter) were installed in the chamber, whose bottom wells were filled with serum-free DMEM with or without histone H1 at various concentrations. Freshly prepared microglia were suspended in serum-free DMEM and were placed into the top wells at 50000 cells/well. The chamber was maintained in a CO _2_ incubator at 37°C for 3 hrs. The filter was removed and stained with RapiDiffII (Biostain RRL, UK), cells on the top side of the filter were wiped off, and the numbers of cells that had migrated to the bottom side were counted as described in ^20^. Four independent experiments were performed (4–8 filters were counted for each condition in each experiment) and the data expressed as cell counts.

### MHC class II assay

Microglial cells were plated at around 50,000 cells/well on glass coverslips. The following day cells were left in serum-free DMEM for 4 hrs and then histone at different concentrations was added to the medium. Twenty-four hours later cells were fixed in 4% (w/v) PFA for 30 min and with ice cold methanol for 5 min. Microglia were labeled using a polyclonal antibody to ionized calcium binding adapter molecule 1 (Iba1) (Wako). For detecting MHC class II antigens we used a monoclonal mouse anti-rat RT1B antibody (OX-6) (Serotec clone 554926), BD Bioscience diluted 1:100. The percentage of cells that were OX-6 positive over the total number of Iba1 positive cells was determined. Three independent experiments, each of them run in triplicate were analysed. Cultures were then treated with lipopolysaccharide (LPS) (Sigma UK) at 1 μg/ml or histone H1 at 25–200 nM for 24 hrs. Primary microglial cultures were incubated in serum-free medium for 24 hrs and identified with Iba1 (red) while reactive microglia were identified with OX-6, which labels MHC-class II expressing cells in green so that double-stained cells appear yellow.

### Survival and death assays

When microglial cells are kept in serum-free conditions and in relatively low concentration (approximately 2000 cells/mm ^2^), apoptotic cell death is observed within 24 hrs ^21^. Therefore, to assess survival, microglia were plated at low density and incubated for 24 hrs in serum-free medium supplemented with increasing doses of histone H1 (50, 100 and 200 nM). Cells were then fixed and immunostained with Iba1 and DAPI. The number of Iba1-stained cells was counted per coverslip in four independent experiments.

The possibility that histone H1 induces apoptosis was tested by staining for activated caspase 3 at 6 and 24 hrs following application of histone H1.

### Statistics

All statistical tests were performed in Sigmaplot v. 12.3 (Systat Software). Either one-way analysis of variance or ANOVA on ranks was used to test differences between groups. All post hoc tests were made with the appropriate Bonferroni/Holm-Sidak correction. Dose-dependent relationships were tested using linear regression where appropriate.

## Results

During the course of experiments using collagen gel co-cultures to identify axonal chemorepellents from different brain regions we observed that adult cortical explants caused embryonic neural explants to degenerate. Having shown that medium conditioned with adult brain explants also caused neurodegeneration, we set about using this system to identify neurotoxic activities and demonstrated that the linker histone H1, but not core histones, caused degeneration of cortical neurons. As neurodegeneration is accompanied by glial activation and subsequently inflammation and activation of the immune system, we investigated whether histone H1 modulates the phenotype of astrocytes and microglia and have also shown that indeed it does.

### Isolation of histones released from mammalian brain

Cultured alone, embryonic explants extend axons vigorously over 24–48 hrs but in the presence of nearby adult brain start to degenerate within 24 hours. We reasoned that this might be caused by diffusible glycosylated proteins or peptides which would bind differentially to lectins. Thus we devised a co-culture assay in which lectin-coated agarose beads were placed between the adult and embryonic explants to see whether degeneration could be inhibited.

**Figure 1.**
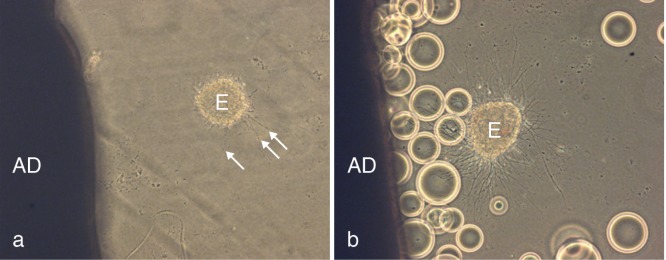
Diffusion-mediated degeneration of adult neocortex is inhibited by *Sambucus nigra* lectin. ( **a**) Shows an adult cortical explant to the left (AD) and a degenerating E 15 embryonic cortical explant (E) to the right; arrows indicate blebbing of degenerating axons. ( **b**) Degeneration is inhibited by placing agarose beads coated with *Sambucus nigra* lectin between the explants.

By trial and error we found that *Sambucus nigra* lectin (SNA)-coated beads greatly reduced or blocked degeneration in collagen gel co-cultures (6/6 experiments, Figure 1). We therefore used SNA-coated beads to construct affinity chromatography columns to adsorb glycosylated moieties which could then be isolated.

Following SDS-PAGE of the column eluates, a variety of proteins was identified by MALDI TOF/TOF/QTOF analysis and included malate dehydrogenase 1, lactate dehydrogenase A, complement component factor h, Ig kappa chain C, ubiquitin-conjugating enzyme E2N, prostaglandin D synthase, prostaglandin D2, serotransferrin precursor protein, glycolipid transfer protein, transferrin, apolipoprotein A, apolipoprotein E and histones, H2A, H2B and H3.

**Figure 2.**
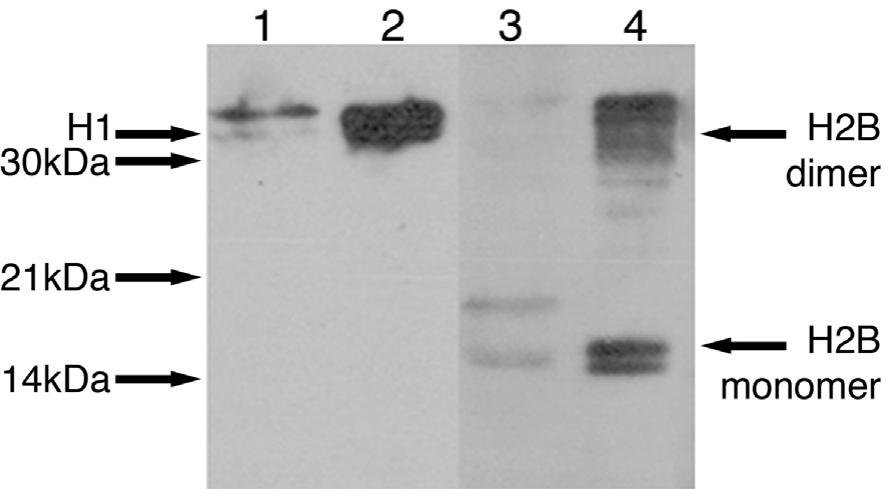
Release of histones H1 and H2B from ischaemic adult brain. Adult conditioned medium (ACM) was made by incubating ischaemic brain slices in defined culture medium that was subjected to affinity chromatography. Western blots using antibodies specific to histones H1 and H2B show the presence of histone H1 at 32 kDa (lane 1) and histone H2B at 16 kDa in ACM (lane 3). Protein standards are shown at 32 kDa (H1 lane 2) and at 16 kDa for the monomer and 32 kDa for the dimer (H2B lane 4).

Having demonstrated the presence of histones in column equates, we found by western blotting that Histones H1 and H2B were present in medium conditioned by adult brain ( Figure 2).

### Histone H1, but not core histones, is neurotoxic

**Figure 3.**
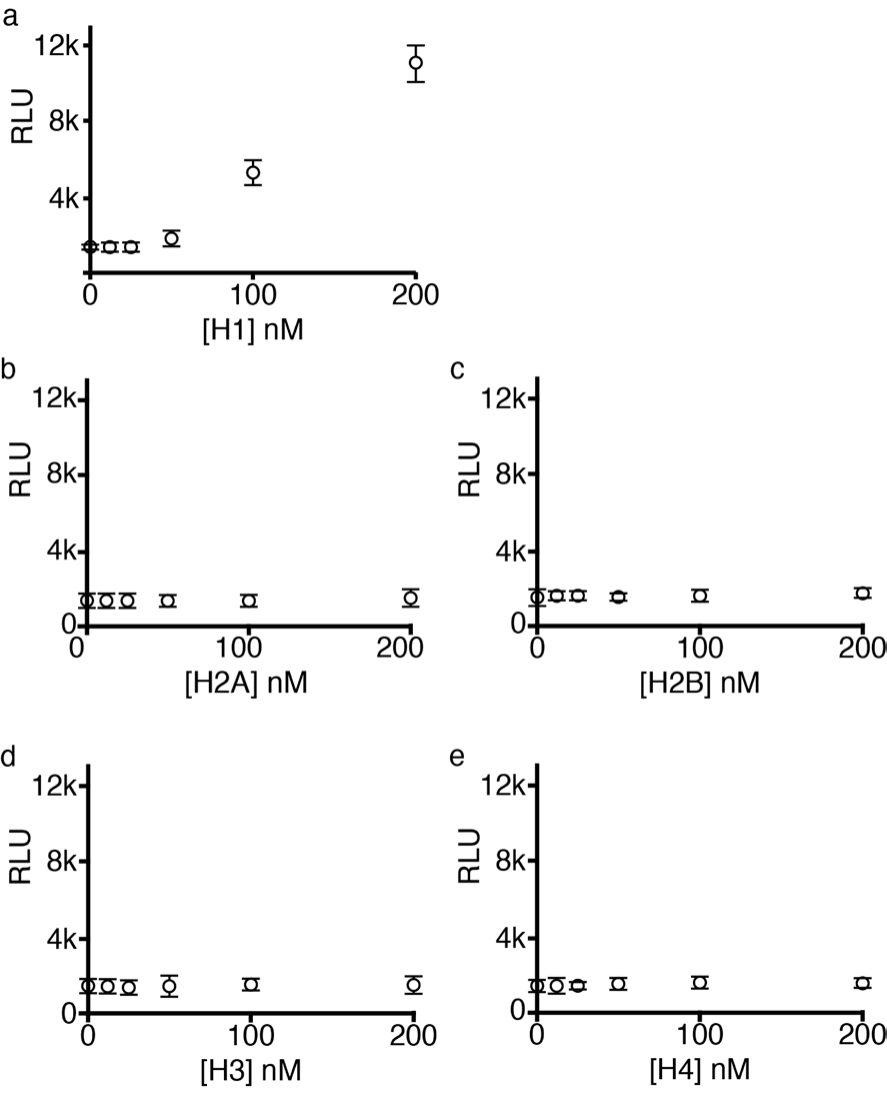
Linker histone H1 is neurotoxic. Dissociated cortical neurons were cultured for 48 hrs and histones were applied for a further 24 hrs. Cell death was determined by luminescence to measure protease release and was expressed as relative luminescence units (RLU). For clarity RLU means are expressed as +/- 2 x standard deviation (SD). Histone H1 caused dose-dependent death of cortical neurons ( **a**) while core histones H2A, H2B, H3 and H4 were without effect up to 200 nM ( **b–e**). Cell death as measured by luminescence was confirmed directly via phase contrast microscopy of dissociated cortical neurons in culture ( Figure 4).

Dissociated embryonic cortical neurons were cultured for 48 hrs and then for a further 24 hrs in the presence of histones H1, H2A, H2B, H3 and H4. Only histone H1 demonstrated neurotoxicity at concentrations below 200 nM ( Figure 3– Figure 5). Histone H1 caused significant degeneration from 100 nM (ANOVA on ranks p<0.001) and a dose-dependent relationship is evident between 0 and 200 nM (R ^2^ = 0.966, n = 6, p<0.001).

**Figure 4.**
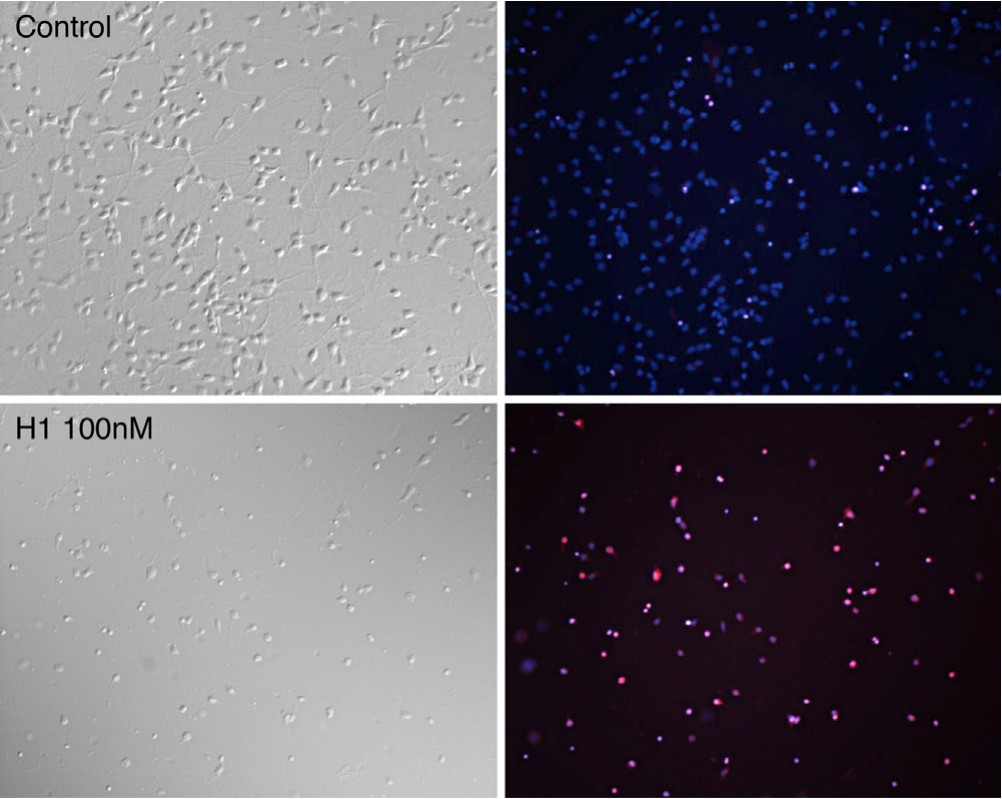
Neurotoxic effects of histone H1 on dissociated cortical neurons. Cortical neurons were plated on laminin-coated coverslips for 48 hrs. Cultures were treated with histone H1 at 50, 100 or 200 nM or left as controls for a further 24 hrs when they were incubated with propidium iodide (PI) for 10 mins prior to fixing and incubation with DAPI. As propidium iodide enters dying cells and binds to nuclear DNA its red fluorescence is greatly enhanced. Top left (control) and lower left (100 nM histone H1) show phase bright examples with their corresponding DAPI (blue) and PI (red) merged images which indicate the numbers of dying cells (magenta) and total nuclei (magenta and blue).

**Figure 5.**
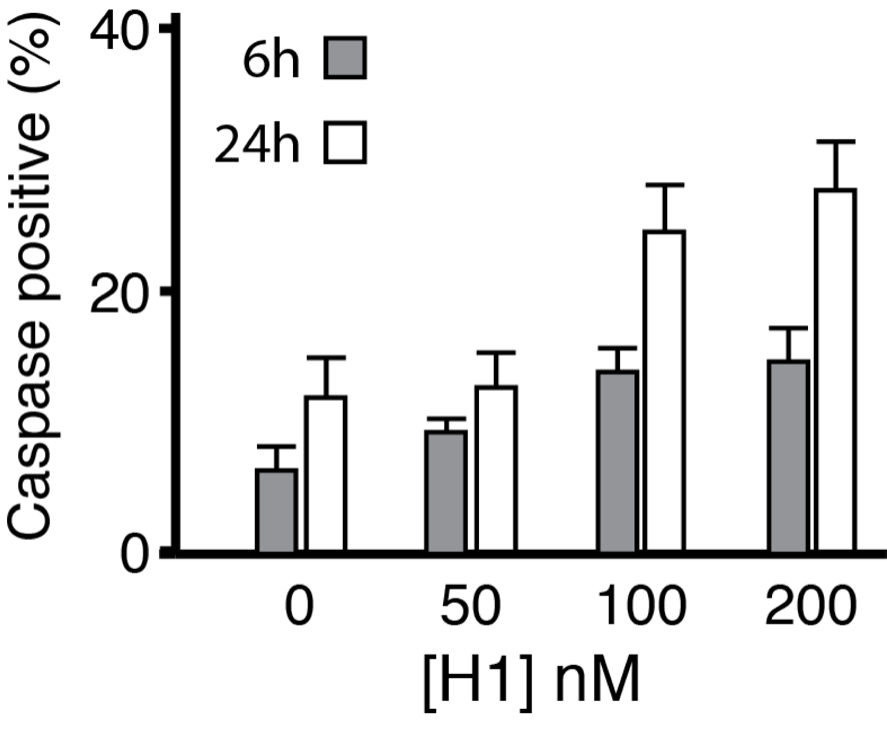
Histone H1 induces apoptosis. Cortical neurons were plated on laminin-coated coverslips and treated with histone H1 at 50, 100 and 200 nM for 6 hrs and 24 hrs. Cells were fixed and stained with DAPI (total nuclei) and for activated caspase 3 to detect apoptosis. Counts for both DAPI and caspase 3 were made for between 11–14 random fields per coverslip for each condition using Image J (University of California San Francisco UCSF) and expressed as % caspase 3/DAPI. Histone H1 induced significant upregulation of activated caspase 3 at 6 and 24 hrs for neurons treated at 100 and 200 nM respectively (ANOVA).

Cell death was determined from protease release and expressed as relative luminescence units (RLU). This was confirmed at a microscopic level and by using the vital dye propidium iodide (which is only taken up by dying cells as their membranes break down) and DAPI which stains all nuclei both in living and dying cells.

**Figure 6.**
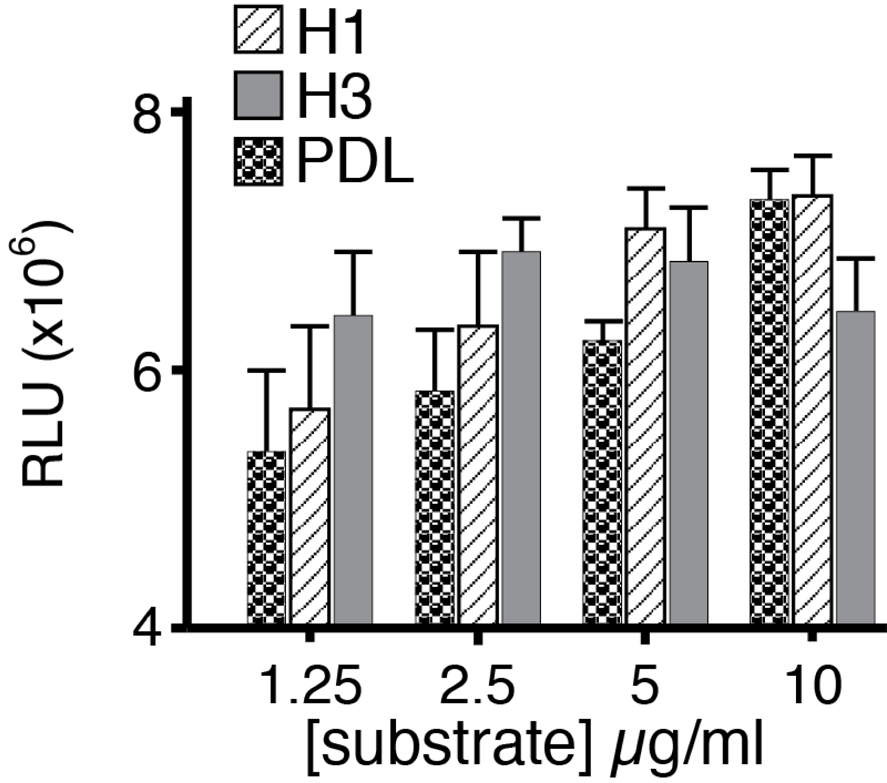
Effects of histones presented in the substratum on neuronal survival. Dissociated neurons were plated on substrate formed by binding histone H1, histone H3, and poly-D lysine to culture dishes at 1.25, 2.5, 5 and 10 μg/ml. Growth on immobilized histone H1 and poly-D-lysine was significantly increased at 5 and 10 μg/ml (one way ANOVA with Bonferroni’s post hoc correction) but was independent of substratum levels of histone H3.

We considered the possibility that the presentation of histones might be a crucial determinant of their actions and investigated the effects of substratum-bound histones on neuronal survival. When bound to the dish however, histone H1 and poly-D-lysine significantly increased neuronal survival compared with controls whereas histone H3 was without effect ( Figure 6).

### Histone H1 induces glial reactivity

**Figure 7.**
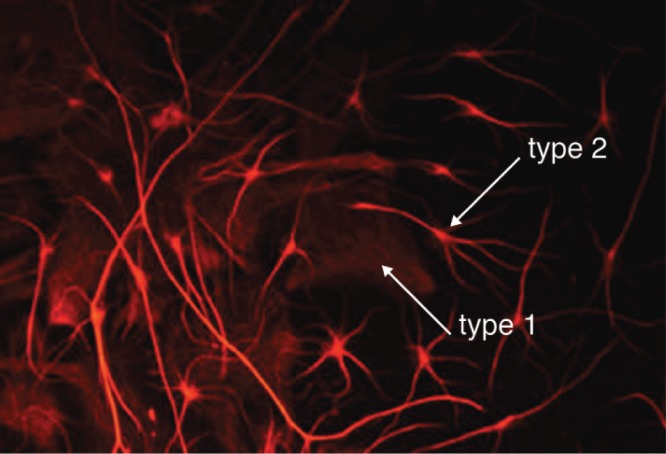
Histone H1 promotes the differentiation of astrocytes. Histone H1 applied for 24 hrs at 50 nM on 6-day cultured cortical astrocytes (visualized using antibodies to GFAP) promoted differentiation of stellate (type 2) over flat (type 1) morphologies.

It is well known that glial cells can proliferate and become activated in regions where neurons are degenerating so we made preliminary qualitative observations of the effects of histone H1 on cortical astrocytes. Control cultures mainly exhibit a flat ‘type 1’ rather than a stellate ‘type 2’ morphology. Histone H1 greatly increased the numbers of astrocytes with a stellate morphology when applied at concentrations as low as 15 nM ( Figure 7). These observations are consistent with a previous report ^23^ and we went on to look at the effects of histone H1 on microglia that mediate the innate immune response of the central nervous system (CNS).

### Histone H1 up-regulates MHC class II expression in microglia

**Figure 8.**
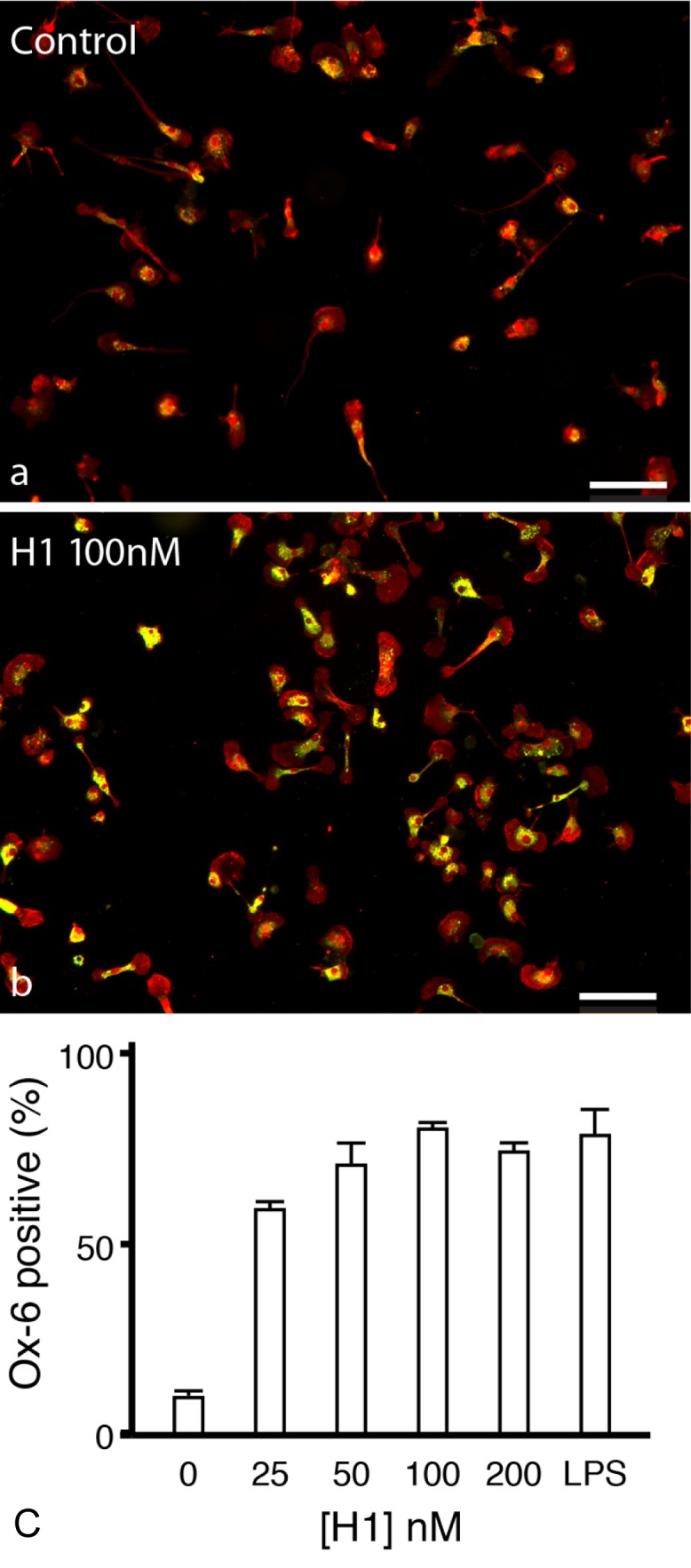
Histone 1 upregulates MHC class II expression in microglia. ( **a**) Primary microglial cultures were incubated in serum-free medium for 24 hrs and identified with Iba1 (red). ( **b**) Cultures were then treated with histone H1 (100 nM) for 24 hrs. Activated microglia were identified with OX-6 (which labels MHC-class II, green) so that double-stained cells appear yellow. Scale bars: 100 μm. ( **c**) Histone H1 dose-dependently increased the expression of MHC class II to a level similar to that elicited by LPS (1 μg/ml). 25 nM H1 produced a 6-fold increase in expression of OX-6 (p<0.001) while the effects of 50 nM histone H1 were not different from the maximal response to LPS at 1 μg/ml. Error bars represent ± SEM for all conditions, one-way ANOVA, Bonferroni post-hoc correction.

Microglial cells are resident monocytes in the CNS and local damage induces them to adopt an effector, or ‘activated’ state, which is associated with the development of an amoeboid morphology, pro-inflammatory cytokine expression and expression of MHC class II antigens ^20^. We looked first at MHC class II expression using OX-6 as a marker following application of histone H1 ( Figure 8a, b) or LPS ( Figure 8b). Around 10% of untreated microglia were OX-6 positive but this increased to 58% when cells were treated with histone H1 at 25 nM and rose to almost 80% with increasing doses such that the effect of 50 nM histone H1 was not different from dosage of LPS which causes a maximal response (8a and b).

### Histone H1 is a potent chemoattractant for microglia

**Figure 9.**
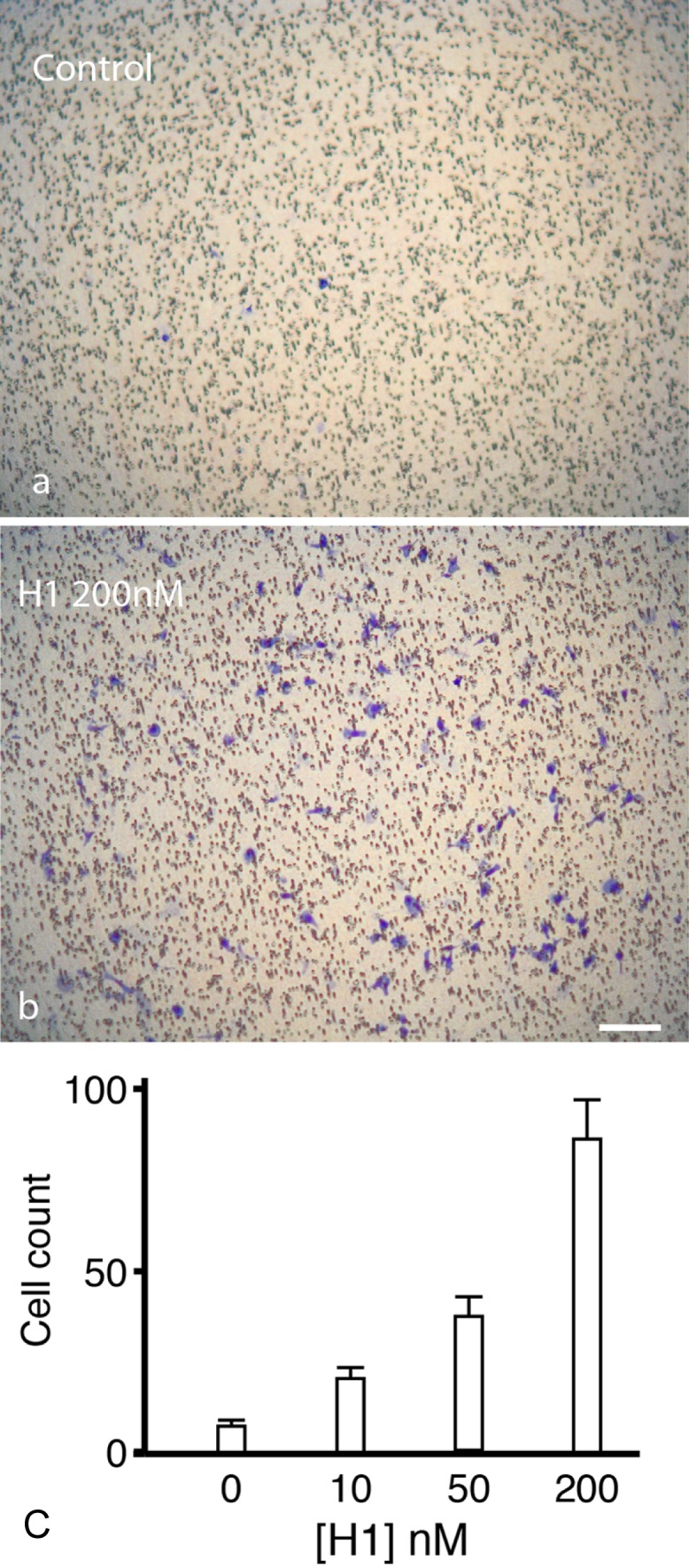
Histone H1 induces microglial chemotaxis. ( **a** and **b**) Chemotaxis towards histone H1 was measured using a Boyden chamber assay and cells that migrated through the filter are labelled in blue. The addition of histone H1 to the lower well of the chamber ( **b**) increased microglial migration to the inner membrane surface compared to controls ( **a**) demonstrating that histone H1 is a potent chemoattractant for microglia. The increases in microglial migration caused by histone H1 were significant (p<0.007 at 50 nM, p<0.001 at 100 and 200 nM one-way ANOVA, Holm-Sidak posthoc correction). Error bars represent ± SEM.

Microglia migrate towards sites of CNS damage that release chemoattractants. Using a Boyden chamber in which microglia migrate up a concentration gradient of attractant through pores in a polycarbonate filter, histone H1 significantly increased chemotaxis in a dose dependent manner ( Figure 9a and b).

### Histone H1 promotes microglial survival

**Figure 10.**
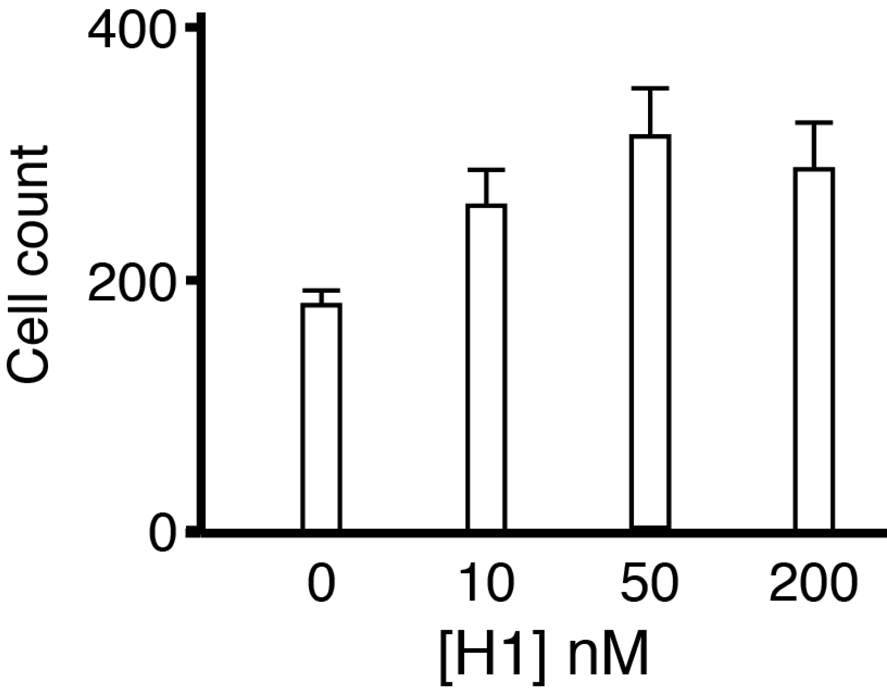
Effects of histone H1 on microglial survival. Treatment with histone H1 increased microglial survival in all the treatment groups considered together (ANOVA p=0.011 Holm-Sidak post hoc correction) and in multiple comparisons survival was increased significantly at both 50 nM and 200 nM H1 compared to control but just failed to reach significance at 10 nM (p=0.06).

Microglial cells were incubated at low concentration in serum-free medium for 24 hrs to assess survival. Previous work indicates that serum deprivation results in a loss of around 50% of microglial cells by 24 hrs ^21^. We quantified the number of cells after 24 hrs of incubation in serum free medium by fixing them and immunostaining with Iba1. For microglia treated with histone H1 at 50 and 200 nM survival was increased significantly but there was no increased effect of histone H1 with increasing dose confirming that in the range over which it is highly toxic to neurons it has the opposite effect on microglia ( Figure 10). We were unable to distinguish between increased longevity and proliferation but experiments were conducted under conditions that lead to cell loss and it seems most plausible that the increased numbers of cells remaining after 24 hrs in culture are due to improved survival rather than increased proliferation.

**Figure 11.**
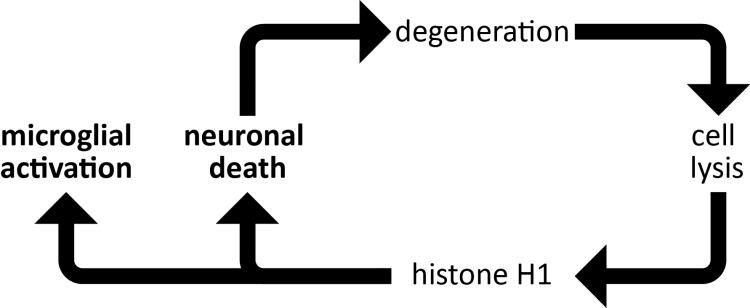
Histone H1 drives neuroinflammation through a positive feedback loop. Histone H1 released from dying neurons causes cell death of neighbouring neurons and simultaneously promotes pro-inflammatory responses in microglia and astrocytes, which may then exacerbate neurodegeneration.

Influence of different histones on neuron cell death and microglial reactivity Histone H1 cell death: Images of dissociated embryonic rat cortical neurons exposed to various concentrations of histone 1 (H1) for 48 hours. Some cultures were stained with either propidium iodide (PI - red) or DAPI (blue) after 24 hours to identify dying cells.Chemotaxis raw data: Images of rat microglia in a Boyden chamber stained with RapiDiffII (blue) at different histone 1 (H1) concentrations. Four independent experiments were performed.Astrocytes + histones: Images of rat cortical astrocytes exposed to either 0 or 50nM of histone 1 (H1) and anti-glial acidic fibrillary protein (GFAP).Ox-6 raw data: Images of rat microglia cultured first stained with Iba1 (red), exposed to either 0 or 200nm of histone 1 (H1) for 24 hours before staining with monoclonal mouse anti-rat RT1B antibody (Ox-6; green) to detect MHC-class II. Double-stained cells appear yellow.Adult cond medium gels: Adult conditioned medium made from rat ischaemic brain slices used to isolate histones.Adult explants + embryonic: Images of adult rat cortical explants and E16 embryonic rat cortical explants. Beads are made from agarose coated with Sambucus nigra lectin. See Figure 2 in the main text for labeling of images.Summary raw data sheets: See ‘Legends for summary raw spreadsheets’ in this folder for descriptions of each spreadsheet in this folder.

## Discussion

### Histone H1 drives neurodegeneration and microglial activation

We show that histone H1 is released from damaged brain explants and that it causes neuronal death and activation of the innate immune response via microglia. Linker histone H1 caused dose-dependent death of cortical neurons and but core histones H2A, H2B, H3 and H4 were without effect up to 200 nM. The effect of histone H1 is therefore selective and argues against histone-induced neurodegeneration being caused by membrane disruption due their highly basic nature. Indeed, histone H1 does not kill astrocytes and microglia but causes them to become reactive. This differential toxicity differs from that observed in sepsis where histones H3 and H4 rather than histone H1 are mediators of endothelial cell death ^13^ and from platelet aggregation where histone H4 is an activator ^14^.

We have shown the release of histones H2A, H2B and H3 from adult brain by sequencing and the release of histones H1 and H2B by western blotting. These observations are thus consistent with previous reports of the extracellular release of histones ^1, 7^ and strengthen the notion that the appearance of histones in the extracellular space can be linked with pathophysiological effects in neighbouring cells.

It has been shown that histone H1 binds directly to polysialic acid and promotes the survival of primary sensory neurons ^23^, an effect opposite to that reported here for soluble histone H1. However, when immobilized on tissue culture plastic histone H1, but not H3, does significantly increase neuronal survival in a dose-dependent manner and is consistent with the effects reported on sensory afferents implying that binding to polysialic acid or plastic masks neurotoxic cell-surface binding or prevents internalization.

We isolated histones using *Sambucus nigra* lectin, which binds sialylated glycoproteins through recognition of Neu5Ac(α2–6)Gal/GalNAc sequences and has particularly high affinity for N-acetylneuraminic acid linked by α2,6-linkages to galactose or N-acetylgalactosamine. Histones have not been reported to be sialylated and it is possible that we co-purified histones bound to sialylated moieties from which they would dissociate under the reducing conditions of SDS PAGE.

### Histone H1 activates innate immunity via microglia

We have shown that low doses of histone H1 dramatically up-regulate the expression of MHC class II antigens, which present pathogen-derived fragments to CD4+ T ‘helper’ cells. Thus, when histone H1 is released from the dying or injured nervous system it drives an inflammatory response via the innate immune system. We also show for the first time that histone H1 is a potent chemoattractant for microglia which are recruited to sites of inflammation and infection by chemokines. Histone H1 also promoted the survival of microglia under stress conditions and taken together these findings are consistent with a novel contribution of extracellular histone H1 to innate immunity in defence of the nervous system. The transition of microglia from a ‘surveying’ (also termed resting) to a pro-inflammatory or ‘effector’ state is widely regarded as a double-edged sword ^24– 26^ within the damaged nervous system and it is now plausible to think that the effects of histone H1 may contribute to this.

### Potential mechanisms of extracellular histone accumulation and toxicity

Little is known of the mechanisms or circumstances by which histone H1 appears in the extracellular space although core histones have been observed in exosomes ^27^. It is possible that there are clearance mechanisms for histones following cell death or apoptosis but that under certain conditions these become overloaded. Histone up-regulation is known to occur in AD, where ectopic non-nuclear H1 is found at the neuronal cell-surface and in activated astrocytes ^28^. Phosphorylated histone H3 is a nuclear marker for the M-phase of normal cell division but becomes activated and re-localised to the cytoplasm of neurons in AD ^29^. There is compelling evidence that aberrant re-entry into the cell cycle may be a significant cause of neuronal cell death in AD ^30^. Moreover, following UV-induced damage, the histone isoform, H1.2, translocates from the nucleus to the cytoplasm and triggers apoptosis ^10^. Thus, one source of extracellular histones including histone H1 ^10, 31^ might be those re-localised to the cytoplasm while another could be those released from disintegrating nuclei. Potentially, extracellular histones could act both at the cell surface and within the cytoplasm following uptake. Hence an accumulation of extracellular histones could exacerbate neurotoxicity and as cell death increases produce an auto-catalytic cascade of neuronal cell death. Histones are also implicated in the formation of insoluble protein deposits. They bind to the Parkinson’s disease-associated protein α-synuclein and increase its fibrillation rate ^32– 34^. Histones also bind Alzheimer β-amyloid precursor protein (APP) and to β-amyloid with high affinity ^32, 35^. Since histones are found within amyloid plaques in AD brains ^36^ an increase in their expression could be a catalyst for neuronal death and would be consistent with the findings of raised anti-histone antibody titres ^12^.

Under cold stress, cortical slices lose their ability to increase respiration in response to electrical stimulation ^37, 38^. This respiratory depression is accompanied by migration of histones from the nucleus into the cytoplasm where they associate with microsomes and mitochondria, which swell. Respiratory depression is most likely to result from inhibition of oxidative metabolism of ATP and is reproduced by addition of soluble histones to the culture medium. Thus, under certain conditions histones can be liberated from the nucleus and taken up with both events leading to neuronal toxicity. These early observations exactly mirror those following UV-induced damage where histone H1.2 translocates from the nucleus to mitochondria via the cytoplasm and triggers apoptosis ^10^. Significantly it has been shown that while linker and core histones bind to mitochondria and release pro-apoptotic proteins only linker histone H1.2 disrupts the inner membrane potential and causes release of the mitochondrial NAD ^+^ pool ^39^. Our observation that histone H1 causes apoptosis via activated caspase 3 is entirely consistent with observations of mitochondrial damage and dysfunction.

Free histones have been reported in blood ^13, 40^ and it is reasonable to expect that their levels would increase, as do those of nucleosomes, following neuronal cell death from whatever cause. It has been known for a considerable time that histones can be cytotoxic to a variety of bacteria and mammalian cells ^13, 41– 43^. Histones may contribute to defence against bacterial and viral infection by acting as cell surface receptors for bacterial and viral proteins and as antimicrobial agents in the gut. Extracellular histones and histone peptides (e.g. ncamp-1, Hipposin, Onchorynciin II, Parasin I, Buforin I/II, MUMP1-3) are non-specific antimicrobial agents of the immune response in fish, amphibians and mammals ^42, 44– 55^. Histones in blood entrap pathogens when neutrophils release parcels of granule proteins and chromatin known as ’neutrophil extracellular traps’ that bind bacteria, fungi and yeast as a part of the innate immune response ^56– 58^. However, as discussed, it may be that the contribution of histone H1 to immune defence in the nervous system is in fact damaging.

We propose that in neurodegenerative diseases and following trauma, histone H1 acts in the brain as an antimicrobial peptide that drives the innate immune system and targets neurons as foreign bodies. We suggest that histones recognise binding sites in the nervous system that are shared with those of bacteria and viruses and that mitochondria, which were evolutionarily derived from bacteria, are the primary intracellular targets of histone H1 within the nervous system.
