# Supplementary material for: Extracellular histone H1 is neurotoxic and drives a pro-inflammatory response in microglia
Source: F1000Res. 2013 Jul 8;2:148. [Version 1] doi: 10.12688/f1000research.2-148.v1 (PMC3782347; doi:10.12688/f1000research.2-148.v1)
Supplement: Influence of different histones on neuron cell death and microglial reactivity — Histone H1 cell death: Images of dissociated embryonic rat cortical neurons exposed to various concentrations of histone 1 (H1) for 48 hours. Some cultures were stained with either propidium iodide (PI - red) or DAPI (blue) after 24 hours to identify dying cells.Chemotaxis raw data: Images of rat microglia in a Boyden chamber stained with RapiDiffII (blue) at different histone 1 (H1) concentrations. Four independent experiments were performed.Astrocytes + histones: Images of rat cortical astrocytes exposed to either 0 or 50nM of histone 1 (H1) and anti-glial acidic fibrillary protein (GFAP).Ox-6 raw data: Images of rat microglia cultured first stained with Iba1 (red), exposed to either 0 or 200nm of histone 1 (H1) for 24 hours before staining with monoclonal mouse anti-rat RT1B antibody (Ox-6; green) to detect MHC-class II. Double-stained cells appear yellow.Adult cond medium gels: Adult conditioned medium made from rat ischaemic brain slices used to isolate histones.Adult explants + embryonic: Images of adult rat cortical explants and E16 embryonic rat cortical explants. Beads are made from agarose coated with Sambucus nigra lectin. See Figure 2 in the main text for labeling of images.Summary raw data sheets: See ‘Legends for summary raw spreadsheets’ in this folder for descriptions of each spreadsheet in this folder. [file f1000research-2-1619-s0000.tgz › Stats.pdf]

Fig 3a H1 – 0 is different to 100 and 200 but not 12.5 or 50

One Way Analysis of Variance

Normality Test (Shapiro-Wilk) Failed (P < 0.050)

Test execution ended by user request, ANOVA on Ranks begun

Kruskal-Wallis One Way Analysis of Variance on Ranks

**Group**	**N**	**Missing**	**Median**	**25%**	**75%**
0.0000	32	0	13557.167	12363.875	15557.688
12.5000	20	0	13895.708	13172.604	15354.021
25.0000	20	0	13938.000	12270.500	16473.750
50.0000	38	0	16685.667	15590.958	22527.859
100.0000	53	0	54308.000	48973.917	58689.906
200.0000	43	0	111201.000	102242.500	117362.667

H = 175.943 with 5 degrees of freedom. (P = <0.001)

The differences in the median values among the treatment groups are greater than would be expected by chance; there is a statistically significant difference (P = <0.001)

To isolate the group or groups that differ from the others use a multiple comparison procedure.

Multiple Comparisons versus Control Group (Dunn's Method) :

**Comparison**	**Diff of Ranks**	**Q**	**P<0.05**
200.0000 vs 0.0000	144.250	10.365	Yes
100.0000 vs 0.0000	96.250	7.212	Yes
50.0000 vs 0.0000	35.803	2.503	No
12.5000 vs 0.0000	8.700	0.512	Do Not Test
25.0000 vs 0.0000	4.400	0.259	Do Not Test

Note: The multiple comparisons on ranks do not include an adjustment for ties.

Fig 3 b-e H2A – no difference

One Way Analysis of Variance

Normality Test (Shapiro-Wilk) Passed (P = 0.895)

Equal Variance Test: Passed (P = 0.617)

Group Name	N	Missing	Mean	Std Dev	SEM
0.0000	32	0	13880.500	1892.466	334.544
12.5000	8	0	14992.625	1484.117	524.715
25.0000	8	0	14798.500	1404.250	496.477
50.0000	8	0	14092.875	1358.809	480.412
100.0000	8	0	14192.625	1586.644	560.964
200.0000	8	0	15695.500	2054.119	726.241

Source of Variation	DF	SS	MS	F	P
Between Groups	5	27158260.361	5431652.072	1.790	0.127
Residual	66	200328391.458	3035278.658		
Total	71	227486651.819			

The differences in the mean values among the treatment groups are not great enough to exclude the possibility that the difference is due to random sampling variability; there is not a statistically significant difference (P = 0.127).

Power of performed test with alpha = 0.050: 0.261

The power of the performed test (0.261) is below the desired power of 0.800.
Less than desired power indicates you are less likely to detect a difference when one actually exists. Negative results should be interpreted cautiously.

H2B – failed normality test therefore anova on ranks 0 versus 200 is different

One Way Analysis of Variance

Normality Test (Shapiro-Wilk) Passed (P = 0.203)

Equal Variance Test: Failed (P < 0.050)

Test execution ended by user request, ANOVA on Ranks begun

Kruskal-Wallis One Way Analysis of Variance on Ranks

Group	N	Missing	Median	25%	75%
0.0000	32	0	13557.167	12363.875	15557.688
12.5000	8	0	15215.000	13670.750	15771.750
25.0000	8	0	15285.000	14144.750	15902.250
50.0000	8	0	14903.500	14220.000	15346.500
100.0000	8	0	15484.000	14241.250	15989.750
200.0000	8	0	16691.500	15159.500	17015.250

H = 16.407 with 5 degrees of freedom. (P = 0.006)

The differences in the median values among the treatment groups are greater than would be expected by chance; there is a statistically significant difference (P = 0.006)

To isolate the group or groups that differ from the others use a multiple comparison procedure.

Multiple Comparisons versus Control Group (Dunn's Method) :

Comparison	Diff of Ranks	Q	P<0.05
**200.0000 vs 0.0000**	**30.188**	**3.649**	**Yes**
25.0000 vs 0.0000	17.188	2.078	No
100.0000 vs 0.0000	15.938	1.927	Do Not Test
12.5000 vs 0.0000	12.313	1.488	Do Not Test
50.0000 vs 0.0000	10.438	1.262	Do Not Test

Note: The multiple comparisons on ranks do not include an adjustment for ties.

H3 – anova no difference

One Way Analysis of Variance

Normality Test (Shapiro-Wilk) Passed (P = 0.553)

Equal Variance Test: Passed (P = 0.709)

Group Name	N	Missing	Mean	Std Dev	SEM
0.0000	32	0	13880.500	1892.466	334.544
12.5000	8	0	14043.500	1733.379	612.842
25.0000	8	0	13876.875	1595.801	564.201
50.0000	8	0	14126.875	2744.915	970.474
100.0000	8	0	15030.125	1603.870	567.054
200.0000	8	0	15403.500	2037.439	720.344

Source of Variation	DF	SS	MS	F	P
Between Groups	5	21404135.250	4280827.050	1.132	0.353
Residual	66	249689381.458	3783172.446		
Total	71	271093516.708			

The differences in the mean values among the treatment groups are not great enough to exclude the possibility that the difference is due to random sampling variability; there is not a statistically significant difference (P = 0.353).

Power of performed test with alpha = 0.050: 0.077

The power of the performed test (0.077) is below the desired power of 0.800.
Less than desired power indicates you are less likely to detect a difference when one actually exists. Negative results should be interpreted cautiously.

H4 – one way anova 0 vs 100 and 0 vs 200 are different

One Way Analysis of Variance

Normality Test (Shapiro-Wilk) Passed (P = 0.946)

Equal Variance Test: Passed (P = 0.568)

Group Name	N	Missing	Mean	Std Dev	SEM
0.0000	32	0	13880.500	1892.466	334.544
12.5000	12	0	14154.583	1989.297	574.261
25.0000	12	0	13980.667	1449.016	418.295
50.0000	12	0	14571.833	1416.573	408.929
100.0000	12	0	15630.250	1585.701	457.752
200.0000	12	0	15524.667	1294.163	373.593

Source of Variation	DF	SS	MS	F	P
Between Groups	5	44808373.051	8961674.610	3.135	0.012
Residual	86	245806561.000	2858215.826		
Total	91	290614934.051			

The differences in the mean values among the treatment groups are greater than would be expected by chance; there is a statistically significant difference (P = 0.012).

Power of performed test with alpha = 0.050: 0.677

Multiple Comparisons versus Control Group (Holm-Sidak method):
Overall significance level = 0.05

Comparisons for factor:

Comparison	Diff of Means	t	P	P<0.050
**0.0000 vs. 100.0000**	**1749.750**	**3.058**	**0.015**	**Yes**
**0.0000 vs. 200.0000**	**1644.167**	**2.873**	**0.020**	**Yes**
0.0000 vs. 50.0000	691.333	1.208	0.544	No
0.0000 vs. 12.5000	274.083	0.479	0.865	No
0.0000 vs. 25.0000	100.167	0.175	0.861	No

100 and 200 nM concentrations appear to show an effect for histone H4, however, comparing these concentrations to the other histones found the values to be similar (see below). Furthermore, H1 shows a greater difference at these concentrations than H4 (see below).

Histone intergroup testing at 200 nM

One Way Analysis of Variance

Normality Test (Shapiro-Wilk) Passed (P = 0.659)

Equal Variance Test: Passed (P = 0.535)

Group Name	N	Missing	Mean	Std Dev	SEM
H2A	8	0	15695.500	2054.119	726.241
H2B	8	0	16302.875	1096.463	387.658
H3	8	0	15403.500	2037.439	720.344
H4	12	0	15524.667	1294.163	373.593

Source of Variation	DF	SS	MS	F	P
Between Groups	3	3977340.764	1325780.255	0.497	0.687
Residual	32	85432999.542	2669781.236		
Total	35	89410340.306			

The differences in the mean values among the treatment groups are not great enough to exclude the possibility that the difference is due to random sampling variability; there is not a statistically significant difference (P = 0.687).

Power of performed test with alpha = 0.050: 0.049

The power of the performed test (0.049) is below the desired power of 0.800.
Less than desired power indicates you are less likely to detect a difference when one actually exists. Negative results should be interpreted cautiously.

H1 versus others at 200 nM

One Way Analysis of Variance

Normality Test (Shapiro-Wilk) Failed (P < 0.050)

Test execution ended by user request, ANOVA on Ranks begun

Kruskal-Wallis One Way Analysis of Variance on Ranks

Group	N	Missing	Median	25%	75%
H1	43	0	111201.000	102242.500	117362.667
H2A	8	0	16622.000	13709.750	17181.750
H2B	8	0	16691.500	15159.500	17015.250
H3	8	0	15712.500	13685.000	17023.750
H4	12	0	15406.500	14370.500	16332.000

H = 58.370 with 4 degrees of freedom. (P = <0.001)

The differences in the median values among the treatment groups are greater than would be expected by chance; there is a statistically significant difference (P = <0.001)

To isolate the group or groups that differ from the others use a multiple comparison procedure.

Multiple Comparisons versus Control Group (Dunn's Method) :

Comparison	Diff of Ranks	Q	P<0.05
H4 vs H1	41.583	5.550	Yes
H3 vs H1	40.875	4.626	Yes
H2A vs H1	38.375	4.343	Yes
H2B vs H1	36.125	4.088	Yes

Note: The multiple comparisons on ranks do not include an adjustment for ties.

Histone intergroup testing at 100 nM

One Way Analysis of Variance

Normality Test (Shapiro-Wilk) Passed (P = 0.425)

Equal Variance Test: Passed (P = 0.671)

Group Name	N	Missing	Mean	Std Dev	SEM
H2A	8	0	14192.625	1586.644	560.964
H2B	8	0	15145.250	987.573	349.160
H3	8	0	15030.125	1603.870	567.054
H4	12	0	15630.250	1585.701	457.752

Source of Variation	DF	SS	MS	F	P
Between Groups	3	9982871.806	3327623.935	1.519	0.228
Residual	32	70114916.500	2191091.141		
Total	35	80097788.306			

The differences in the mean values among the treatment groups are not great enough to exclude the possibility that the difference is due to random sampling variability; there is not a statistically significant difference (P = 0.228).

Power of performed test with alpha = 0.050: 0.140

The power of the performed test (0.140) is below the desired power of 0.800.
Less than desired power indicates you are less likely to detect a difference when one actually exists. Negative results should be interpreted cautiously.

Histone H1 versus other groups at 100 nM

One Way Analysis of Variance

Normality Test (Shapiro-Wilk) Failed ($P < 0.050$)

Test execution ended by user request, ANOVA on Ranks begun

Kruskal-Wallis One Way Analysis of Variance on Ranks

Group	N	Missing	Median	25%	75%
H1	53	0	54308.000	48973.917	58689.906
H2A	8	0	14409.500	13344.250	15602.000
H2B	8	0	15484.000	14241.250	15989.750
H3	8	0	15145.500	13703.000	16323.250
H4	12	0	16067.000	14232.500	16611.750

H = 64.296 with 4 degrees of freedom. ($P = <0.001$)

The differences in the median values among the treatment groups are greater than would be expected by chance; there is a statistically significant difference ($P = <0.001$)

To isolate the group or groups that differ from the others use a multiple comparison procedure.

Multiple Comparisons versus Control Group (Dunn's Method) :

Comparison	Diff of Ranks	Q	P<0.05
H2A vs H1	50.500	5.153	Yes
H2B vs H1	44.375	4.528	Yes
H3 vs H1	44.375	4.528	Yes
H4 vs H1	40.667	4.924	Yes

Note: The multiple comparisons on ranks do not include an adjustment for ties.

Fig 5 Caspase

After 6 hour treatment 100 and 200 but not 50 are different to control

One Way Analysis of Variance

Data source: Capsase 6 vs 24

Normality Test (Shapiro-Wilk) Passed (P = 0.839)

Equal Variance Test: Passed (P = 0.260)

Group Name	N	Missing	Mean	Std Dev	SEM
CON 6	10	0	6.322	5.840	1.847
H1 50 at 6	10	0	9.323	3.138	0.992
H1 100 at 6	14	0	13.803	6.457	1.726
H1 200 at 6	10	0	14.724	7.594	2.401

Source of Variation	DF	SS	MS	F	P
Between Groups	3	491.790	163.930	4.502	0.008
Residual	40	1456.610	36.415		
Total	43	1948.400			

The differences in the mean values among the treatment groups are greater than would be expected by chance; there is a statistically significant difference (P = 0.008).

Power of performed test with alpha = 0.050: 0.741

Multiple Comparisons versus Control Group (Holm-Sidak method):
Overall significance level = 0.05

Comparisons for factor:

Comparison	Diff of Means	t	P	P<0.050
CON 6 vs. H1 200 at 6	8.401	3.113	0.010	Yes
CON 6 vs. H1 100 at 6	7.481	2.994	0.009	Yes
CON 6 vs. H1 50 at 6	3.000	1.112	0.273	No

After 24 hour treatment 100 and 200 but not 50 are different to control

One Way Analysis of Variance

Data source: Capsase 6 vs 24

Normality Test (Shapiro-Wilk) Failed (P < 0.050)

Test execution ended by user request, ANOVA on Ranks begun

Kruskal-Wallis One Way Analysis of Variance on Ranks

Data source: Capsase 6 vs 24

**Group**	**N**	**Missing**	**Median**	**25%**	**75%**
CON 24	9	0	8.511	5.461	19.989
H1 50 at 24	10	0	11.555	3.655	21.344
H1 100 at 24	10	0	21.325	12.872	38.630
H1 200 at 24	12	0	21.548	20.849	34.534

H = 13.599 with 3 degrees of freedom. (P = 0.004)

The differences in the median values among the treatment groups are greater than would be expected by chance; there is a statistically significant difference (P = 0.004)

To isolate the group or groups that differ from the others use a multiple comparison procedure.

Multiple Comparisons versus Control Group (Dunn's Method):

**Comparison**	**Diff of Ranks**	**Q**	**P<0.05**
H1 200 at 24 vs CON 24	15.806	2.992	Yes
H1 100 at 24 vs CON 24	13.022	2.366	No
H1 50 at 24 vs CON 24	1.722	0.313	Do Not Test

Note: The multiple comparisons on ranks do not include an adjustment for ties.

Individual t-test on the data confim that 50 is not different to control after either 6 or 24 hours

t-test

Data source: Capsase 6 vs 24

Normality Test (Shapiro-Wilk) Failed (P < 0.050)

Test execution ended by user request, Rank Sum Test begun

Mann-Whitney Rank Sum Test

Data source: Capsase 6 vs 24

**Group**	**N**	**Missing**	**Median**	**25%**	**75%**
CON 6	10	0	3.956	2.197	10.789
H1 50 at 6	10	0	8.791	6.312	13.201

Mann-Whitney U Statistic= 25.000

T = 80.000 n(small)= 10 n(big)= 10 (P = 0.064)

The difference in the median values between the two groups is not great enough to exclude the possibility that the difference is due to random sampling variability; there is not a statistically significant difference (P = 0.064)

t-test

Data source: Capsase 6 vs 24

Normality Test (Shapiro-Wilk)   Passed   (P = 0.107)

Equal Variance Test: Passed   (P = 0.875)

**Group Name**	**N**	**Missing**	**Mean**	**Std Dev**	**SEM**
CON 24	9	0	11.889	8.624	2.875
H1 50 at 24	10	0	12.627	8.051	2.546

Difference   -0.737
t = -0.193 with 17 degrees of freedom.

95 percent two-tailed confidence interval for difference of means: -8.808 to 7.334

Two-tailed P-value = 0.849

The difference in the mean values of the two groups is not great enough to reject the possibility that the difference is due to random sampling variability. There is not a statistically significant difference between the input groups (P = 0.849).

One-tailed P-value = 0.425

The sample mean of group H1 50 at 24   does not exceed the sample mean of the group CON 24 by an amount great enough to exclude the possibility that the difference is due to random sampling variability. The hypothesis that the population mean of group CON 24 is greater than or equal to the population mean of group H1 50 at 24 cannot be rejected. (P = 0.425).

Power of performed two-tailed test with alpha = 0.050: 0.054

The power of the performed test (0.054) is below the desired power of 0.800.
Less than desired power indicates you are less likely to detect a difference when one actually exists. Negative results should be interpreted cautiously.

Fig 6 Substrates

PDL – 5 and 10 but not 2.5 is different to 1.25

One Way Analysis of Variance

Data source: HV111S

Normality Test (Shapiro-Wilk) Failed (P < 0.050)

Test execution ended by user request, ANOVA on Ranks begun

Kruskal-Wallis One Way Analysis of Variance on Ranks

Data source: HV111S

Group	N	Missing	Median	25%	75%
1.2500	12	0	5385972.000	4935017.000	5791067.500
2.5000	12	0	5789869.500	5599905.000	6157711.750
5.0000	12	0	6179598.000	6083917.250	6381019.250
10.0000	12	0	7329992.000	7172166.250	7418167.000

H = 34.679 with 3 degrees of freedom. (P = <0.001)

The differences in the median values among the treatment groups are greater than would be expected by chance; there is a statistically significant difference (P = <0.001)

To isolate the group or groups that differ from the others use a multiple comparison procedure.

Multiple Comparisons versus Control Group (Dunn's Method):

Comparison	Diff of Ranks	Q	P<0.05
10.0000 vs 1.2500	31.750	5.555	Yes
5.0000 vs 1.2500	16.333	2.858	Yes
2.5000 vs 1.2500	6.917	1.210	No

Note: The multiple comparisons on ranks do not include an adjustment for ties.

PDL – or run the means and all are different to 1.25.

One Way Analysis of Variance

Data source: PDL

Normality Test (Shapiro-Wilk) Failed (P < 0.050)

Equal Variance Test: Failed (P < 0.050)

Group Name	N	Missing	Mean	Std Dev	SEM
1.2500	12	0	5366390.417	608827.919	175753.482
2.5000	12	0	5799679.583	469190.202	135443.545
5.0000	12	0	6205013.417	167204.644	48267.823
10.0000	12	0	7297945.833	196843.677	56823.875

Source of Variation	DF	SS	MS	F	P
Between Groups	3	2.468E+013	8.226E+012	50.040	<0.001
Residual	44	7.233E+012	164378926934.464		

Total	47	3.191E+013

The differences in the mean values among the treatment groups are greater than would be expected by chance; there is a statistically significant difference (P = <0.001).

Power of performed test with alpha = 0.050: 1.000

Multiple Comparisons versus Control Group (Holm-Sidak method):
Overall significance level = 0.05

Comparisons for factor:

**Comparison**	**Diff of Means**	**t**	**P**	**P<0.050**
1.2500 vs. 10.0000	1931555.417	11.670	<0.001	Yes
1.2500 vs. 5.0000	838623.000	5.067	<0.001	Yes
1.2500 vs. 2.5000433289.167		2.618	0.012	Yes

H1 all are different to 1.25

One Way Analysis of Variance

Data source: H1

Normality Test (Shapiro-Wilk) Passed (P = 0.901)

Equal Variance Test: Passed (P = 0.586)

**Group Name**	**N**	**Missing**	**Mean**	**Std Dev**	**SEM**
1.2500	12	0	5209347.917	258936.421	74748.506
2.5000	12	0	6045009.583	205111.454	59210.577
5.0000	12	0	7084854.333	323293.841	93326.893
10.0000	12	0	7527943.750	302330.631	87275.336

**Source of Variation**	**DF**	**SS**	**MS**	**F**	**P**
Between Groups	3	3.921E+013	1.307E+013	171.366	<0.001
Residual	44	3.355E+012	76260374224.517		
Total	47	4.256E+013			

The differences in the mean values among the treatment groups are greater than would be expected by chance; there is a statistically significant difference (P = <0.001).

Power of performed test with alpha = 0.050: 1.000

Multiple Comparisons versus Control Group (Holm-Sidak method):
Overall significance level = 0.05

Comparisons for factor:

**Comparison**	**Diff of Means**	**t**	**P**	**P<0.050**
1.2500 vs. 10.0000	2318595.833	20.566	<0.001	Yes
1.2500 vs. 5.0000	1875506.417	16.636	<0.001	Yes
1.2500 vs. 2.5000	835661.667	7.412	<0.001	Yes

H1 and H1A all different with means

One Way Analysis of Variance

Data source: H1 H11A

Normality Test (Shapiro-Wilk) Failed (P < 0.050)

Equal Variance Test: Passed (P = 0.562)

Group Name	N	Missing	Mean	Std Dev	SEM
1.2500	12	0	6206270.000	505972.812	146061.770
2.5000	12	0	6698473.417	625713.081	180627.808
5.0000	12	0	7131414.917	303490.879	87610.270
10.0000	12	0	7190751.333	264889.008	76466.870

Source of Variation	DF	SS	MS	F	P
Between Groups	3	7.502E+012	2.501E+012	12.352	<0.001
Residual	44	8.908E+012	202449561694.057		
Total	47	1.641E+013			

The differences in the mean values among the treatment groups are greater than would be expected by chance; there is a statistically significant difference (P = <0.001).

Power of performed test with alpha = 0.050: 0.999

Multiple Comparisons versus Control Group (Holm-Sidak method):
Overall significance level = 0.05

Comparisons for factor:

Comparison	Diff of Means	t	P	P<0.050
1.2500 vs. 10.0000	984481.333	5.360	<0.001	Yes
1.2500 vs. 5.0000	925144.917	5.036	<0.001	Yes
1.2500 vs. 2.5000	492203.417	2.680	0.010	Yes

H1 and H11A – all but the 2.5 group different with medians

One Way Analysis of Variance

Data source: H1 H11A

Normality Test (Shapiro-Wilk) Failed (P < 0.050)

Test execution ended by user request, ANOVA on Ranks begun

Kruskal-Wallis One Way Analysis of Variance on Ranks

Data source: H1 H11A

Group	N	Missing	Median	25%	75%
1.2500	12	0	6257470.500	5734357.500	6686506.000
2.5000	12	0	6886542.000	6702693.750	7003098.750
5.0000	12	0	7163226.500	6970030.000	7326806.500
10.0000	12	0	7268587.500	6969436.750	7434899.750

H = 26.623 with 3 degrees of freedom. (P = <0.001)

The differences in the median values among the treatment groups are greater than would be expected by chance; there is a statistically significant difference (P = <0.001)

To isolate the group or groups that differ from the others use a multiple comparison procedure.

Multiple Comparisons versus Control Group (Dunn's Method) :

**Comparison**	**Diff of Ranks**	**Q**	**P<0.05**
10.0000 vs 1.2500	25.583	4.476	Yes
5.0000 vs 1.2500	24.167	4.228	Yes
2.5000 vs 1.2500	11.250	1.968	No

Note: The multiple comparisons on ranks do not include an adjustment for ties.

HV111S only 2.5 is different – but not convincing seems odd quirk of stretch 1.25 group

One Way Analysis of Variance

Data source: HV111S

Normality Test (Shapiro-Wilk) Passed (P = 0.631)

Equal Variance Test: Passed (P = 0.241)

**Group Name**	**N**	**Missing**	**Mean**	**Std Dev**	**SEM**
1.2500	12	1	6248762.182	478377.569	144236.264
2.5000	12	0	6696113.167	266833.643	77028.238
5.0000	12	0	6624321.583	423301.392	122196.586
10.0000	12	0	6269714.167	395012.240	114030.211

**Source of Variation**	**DF**	**SS**	**MS**	**F**	**P**
Between Groups	3	1.918E+012	639172169387.812	4.066	0.013
Residual	43	6.759E+012	157187423948.928		
Total	46	8.677E+012			

The differences in the mean values among the treatment groups are greater than would be expected by chance; there is a statistically significant difference (P = 0.013).

Power of performed test with alpha = 0.050: 0.677

Multiple Comparisons versus Control Group (Holm-Sidak method):
Overall significance level = 0.05

Comparisons for factor:

**Comparison**	**Diff of Means**	**t**	**P**	**P<0.050**
1.2500 vs. 2.5000	447350.985	2.703	0.029	Yes
1.2500 vs. 5.0000	375559.402	2.269	0.056	No
1.2500 vs. 10.0000	20951.985	0.127	0.900	No

Figure 8b Ox-6 stats

One Way Analysis of Variance

Group Name	N	Missing	Mean	Std Dev	SEM
Row 1	8	0	9.342	5.364	1.896
Row 2	9	0	58.332	6.341	2.114
Row 3	9	0	69.797	18.884	6.295
Row 4	9	0	79.085	12.180	4.060
Row 5	9	0	73.784	6.499	2.166
Row 6	9	0	77.588	21.008	7.003

Source of Variation	DF	SS	MS	F	P
Between Groups	5	28910.148	5782.030	32.231	<0.001
Residual	47	8431.461	179.393		
Total	52	37341.609			

The differences in the mean values among the treatment groups are greater than would be expected by chance; there is a statistically significant difference (P = <0.001).

Power of performed test with alpha = 0.050: 1.000

All Pairwise Multiple Comparison Procedures (Holm-Sidak method):
Overall significance level = 0.05

Comparisons for factor:

Comparison	Diff of Means	t	P	P<0.050
Row 4 vs. Row 1	69.743	10.716	<0.001	Yes
Row 6 vs. Row 1	68.246	10.486	<0.001	Yes
Row 5 vs. Row 1	64.442	9.902	<0.001	Yes
Row 3 vs. Row 1	60.455	9.289	<0.001	Yes
Row 2 vs. Row 1	48.990	7.527	<0.001	Yes
Row 4 vs. Row 2	20.753	3.287	0.019	Yes
Row 6 vs. Row 2	19.256	3.050	0.033	Yes
Row 5 vs. Row 2	15.452	2.447	0.137	No
Row 3 vs. Row 2	11.465	1.816	0.424	No
Row 4 vs. Row 3	9.289	1.471	0.617	No
Row 6 vs. Row 3	7.792	1.234	0.717	No
Row 4 vs. Row 5	5.301	0.840	0.875	No
Row 5 vs. Row 3	3.988	0.632	0.897	No
Row 6 vs. Row 5	3.804	0.602	0.797	No
Row 4 vs. Row 6	1.497	0.237	0.814	No

Chemotaxis Figure 9b

One Way Analysis of Variance

Normality Test (Shapiro-Wilk) Failed (P < 0.050)

Equal Variance Test: Failed (P < 0.050)

**Group Name**	**N**	**Missing**	**Mean**	**Std Dev**	**SEM**
con	20	0	7.350	5.174	1.157
10nM	24	0	20.000	15.668	3.198
50nM	22	0	37.591	25.271	5.388
200nM	24	0	85.583	52.951	10.809

**Source of Variation**	**DF**	**SS**	**MS**	**F**	**P**
Between Groups	3	80822.254	26940.751	27.565	<0.001
Residual	86	84053.702	977.369		
Total	89	164875.956			

The differences in the mean values among the treatment groups are greater than would be expected by chance; there is a statistically significant difference (P = <0.001).

Power of performed test with alpha = 0.050: 1.000

All Pairwise Multiple Comparison Procedures (Holm-Sidak method):
Overall significance level = 0.05

Comparisons for factor:

**Comparison**	**Diff of Means**	**t**	**P**	**P<0.050**
200nM vs. con	78.233	8.265	<0.001	Yes
200nM vs. 10nM	65.583	7.267	<0.001	Yes
200nM vs. 50nM	47.992	5.201	<0.001	Yes
50nM vs. con	30.241	3.131	0.007	Yes
50nM vs. 10nM	17.591	1.906	0.116	No
10nM vs. con	12.650	1.336	0.185	No

Fig 10 Microglia survival – Groups are different 50 to 0 and 200 to 0. 10 to 0 trends to show an increase in the treatment group.

Normality Test (Shapiro-Wilk) Passed (P = 0.084)

Equal Variance Test: Failed (P < 0.050)

Group Name	N	Missing	Mean	Std Dev	SEM
0 nM	15	0	179.733	52.009	13.429
10nM	14	0	259.357	114.494	30.600
50nM	13	0	316.385	138.491	38.410
200nM	15	0	291.667	125.355	32.366

Source of Variation	DF	SS	MS	F	P
Between Groups	3	153018.004	51006.001	4.106	0.011
Residual	53	658434.558	12423.294		
Total	56	811452.561			

The differences in the mean values among the treatment groups are greater than would be expected by chance; there is a statistically significant difference (P = 0.011).

Power of performed test with alpha = 0.050: 0.692

Multiple Comparisons versus Control Group (Holm-Sidak method):
Overall significance level = 0.05

Comparisons for factor:

Comparison	Diff of Means	t	P	P<0.050
0 nM vs. 50nM	136.651	3.235	0.006	Yes
0 nM vs. 200nM	111.933	2.750	0.016	Yes
0 nM vs. 10nM	79.624	1.922	0.060	No
