# Supplementary material for: Extracellular histone H1 is neurotoxic and drives a pro-inflammatory response in microglia
Source: F1000Res. 2013 Jul 8;2:148. [Version 1] doi: 10.12688/f1000research.2-148.v1 (PMC3782347; doi:10.12688/f1000research.2-148.v1)
Supplement: Peer review history

# Peer review: Referee response for version 1

**Published:** 2013-07-30

**Authors:** W Sue Griffin

**Affiliations:** 1 University of Arkansas for Medical Sciences, Little Rock, AR, USA

I think this paper excellent and is an important addition to the literature. I really like the conceptualization of a self-replicating cycle as it illustrates the concept that the “problem” starts with the neuron, i.e., due to one or more of a variety of insults, the neuron is negatively impacted and releases H1, which in turn activates microglia with over expression of cytokines that may, when limited, foster repair but when activated becomes chronic (as is demonstrated here with the potential of cyclic H1 release) and thus facilitates neurotoxicity.  I hope the authors intend to measure cytokine expression soon, especially IL-1 and TNF in both astrocytes and microglia, and S100B in astrocytes.

**02/08/2013 - additional comments**

In more detail, Gilthorpe and colleagues provide novel experimental data that demonstrate a new role for a specific histone protein—the linker histone, H1—in neurodegeneration. This study, which was originally designed to identify axonal chemorepellents, actually provided a previously unknown role for H1, as well as other novel and thought provoking results. Fortuitously, as sometimes happens, the authors had a pleasant surprise: their results set some old dogmas on their respective ears and opened up new avenues of approach for studying the role of histones in self-amplification of neurodegenerative cycles. In point, they show that H1 is not just a nice little partner of nuclear DNA as previously thought. H1 is released from ‘damaged’ (or leaky) neurons, kills adjacent healthy neurons, and promotes a proinflammatory profile in both microglia and astrocytes. 

Interestingly, the authors’ conceptualization of a damaged neuron → H1 release → healthy neuron killing cycle does not take into account the H1-mediated proinflammatory glial response. This facet of the study opens for these investigators a new avenue they may wish to follow: the role of H1 in stimulation of neuroinflammation with overexpression of cytokines. This is interesting, as neuronal injury has been shown to set in motion an acute phase response that activates glia, increases their expression of cytokines (interleukin-1 and S100B), which, in turn, induce neurons to produce excess Alzheimer-related proteins such as βAPP and ApoE (favoring formation of mature Aβ/ApoE plaques), activated MAPK-p38 and hyperphosphorylated tau (favoring formation of neurofibrillary tangles), and α synuclein (favoring formation of Lewy bodies). To date, the neuronal response shown responsible for stimulating glia is neuronal stress related release of sAPP, but these H1 results from Gilthorpe and colleagues may contribute to or exacerbate the role of sAPP.

I have read this submission. I believe that I have an appropriate level of expertise to confirm that it is of an acceptable scientific standard.

# Peer review: Referee response for version 1

**Published:** 2013-07-15

**Authors:** Jan-Marino Ramirez

**Affiliations:** 1 University of Washington, Seattle, WA, USA

This is a fantastic paper and a very important contribution to our understanding of neurodegenerative disease and the response of the brain to injury. The authors have done a marvellous job in demonstrating that the adult brain releases histone H1 that were neurotoxic. The toxicity was specific for the H1 histone, while no toxicity was seen for H2, H2B, H3 and H4. An exciting finding is that H1, while neurotoxic to cortical cells promotes the survival of microglia, and acts as a potent chemoattractant; conceptually this is an important finding. 

The study is well done, the conclusions are fair and balanced and the techniques used in the study are very rigorous. The final figure nicely summarizes the proposed role of histones in inducing neuronal cell death whilst activating microglia. I am confident that this study will be a much cited contribution to the field of traumatic brain injury. The work is well written and the figures are outstanding.

I have read this submission. I believe that I have an appropriate level of expertise to confirm that it is of an acceptable scientific standard.

# Peer review: Referee response for version 1

**Published:** 2013-07-12

**Authors:** Roger Keynes

**Affiliations:** 1 University of Cambridge, Cambridge, UK

I think this is a very nice paper. This interesting study identifies a novel mechanism to explain the differential susceptibilities of neurons and glia in neurodegenerative disease and injury, and so makes a significant advance. *In vitro* experiments implicate Histone H1, but not core histones, as selectively toxic for neurons while simultaneously activating astrocytes and microglia. The data are convincing and the proposed H1 positive feedback model (Figure 11) warrants future investigation *in vivo*.

**Suggested revisions**:

**Figure 1**

- The caption should read 'Diffusion-mediated degeneration of embryonic neocortex is...'
- The blebbing of degenerating axons is indistinct as shown and would be more convincing at a higher magnification.
- It would also be interesting to know whether neuronal cell bodies, and not just their axons, are susceptible to H1 toxicity in the collagen gel co-culture; and to confirm that H1-antibody-coated beads pull off the neurotoxic activity.

**Figure 7**

- The type 1 morphology is indistinct - it would be helpful to show or state the typical appearance of astrocytes without added H1.

I have read this submission. I believe that I have an appropriate level of expertise to confirm that it is of an acceptable scientific standard.

# Peer review: Referee response for version 1

**Published:** 2013-07-08

**Authors:** Alain Prochiantz

**Affiliations:** 1 Collège de France, Paris, Paris, France

Very convincing. It would be nice to know if neutralizing H1 in the culture medium modifies axon degeneration and/or cell death.

I have read this submission. I believe that I have an appropriate level of expertise to confirm that it is of an acceptable scientific standard.
